# Have We Forgotten the Threat Posed by Fascioliasis? A Potential Threat to Public Health

**Published:** 2020-04

**Authors:** Wali KHAN, Mudassar IQBAL, Omer DAD

**Affiliations:** 1. Laboratory of Parasitology, Department of Zoology, University of Malakand, Lower Dir, Pakistan; 2. Department of Zoology, Hazara University, Mansehra, Khyber Pakhtunkhwa, Pakistan

## Dear Editor-in-Chief

Fascioliasis is a zoonotic disease, still a critical public health problem in endemic areas. Occurrence of fascioliasis in man is prehistoric (1). The main cause of this disease is *Fasciola hepatica* (the sheep live fluke or the common liver fluke). This flatworm involves biliary passages of the liver, but can also be found in the duodenum, migrate through the intestinal wall into the peritoneal cavity, penetrate the capsule of the liver, pass through its parenchyma and ultimately settle in the biliary passages. As a public health hazards, fascioliasis was first discovered by Chen and Mott (2). Intestinal parasitic infections are widely prevalent in human population of Pakistan with only two reports on *F. hepatica* infection. First report on the occurrence of *F. hepatica* was claimed by Qureshi et al (3) and recently another case is given (4) from Swat, Pakistan. We herein, report the effect of albendazole against fascioliasis in a 14 yr old boy from February to June 2016 of the University Pubic School, University of Malakand, Lower Dir, Khyber Pakhtunkhwa, Pakistan.

A 14-yr boy presented to Laboratory of Parasitology, Department of Zoology, University of Malakand. During the physical examination, no symptoms were observed. Two stool samples were collected from the patient one before diagnosing and one after treatment. The samples were fixed in 10% formalin and tested by light microscopy using the methods of direct smear in Lugol’s solution, normal saline solution and flotation techniques. The microscopic examination of the stool samples demonstrated the presence of *Fasciola hepatica* infection with *Ascaris lumbricoides,* hookworm and *Hymenolepis nana*. The shape of the ovum was oval, opperculated at one end, brownish in color, measuring 120 by 70 micron having an unsegmented ovum with a mass of yolk cells. The number of eggs, for the infection, was calculated prior to diagnosing and after treatment. The treatment was done with albendazole 400 mg, 3 times a day for 7 d. The efficacy [cure rates (CR)] at post-treatment was calculated. Albendazole was continued for 3 months. No infection with *F. hepatica* was observed on the 4th month of follow-up.

Albendazole is widely used against helminth parasitic diseases, its efficacy was 75% when it was used (5). A study conducted at Egypt (6) on the efficacy of albendazole had high efficacy against fascioliasis. In present research, albendazole was used for the treatment of fascioliasis and its efficacy was 100%. Complications such as *F. hepatica* involves in biliary tract and causes common bile duct obstruction are prevented by surgery. Prednisolone at a dose of 10–20 mg/day is used to tackle toxoaemia. Antibiotics are recommended to control secondary bacterial infection.

However, what makes our case unique, is the inclusion of drug used for the treatment of the disease in the drug-list of Pakistan.

**Fig. 1:**
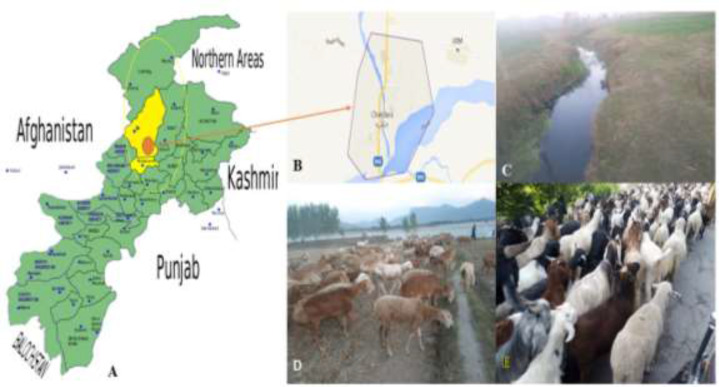
Human infection risk: A) Location of District Malakand, District Lower Dir and Swat (encircled with yellow colour) in KP Province Pakistan: B) View of the locality, C) Source of water collection from an irrigational water channel, (D&E) the overflow of water on the surface of ground where the snail vector species can survive and evidenced to infect the grazing animals. Human becomes infected after eating aquatic plants on which cercariae larvae are encysted or by drinking such contaminated water.

Particularly in endemic areas, in patients presenting with a complaint of fascioliasis, risk factors (Fig. 1) and drug therapy should be taken into consideration. The routine examination of feces is recommended for *F. hepatica* eggs, which may be helpful to make a correct diagnosis.
